# Optimization of seeding density of OP9 cells to improve hematopoietic differentiation efficiency

**DOI:** 10.1186/s12860-024-00503-x

**Published:** 2024-03-25

**Authors:** Xin-xing Jiang, Meng-yi Song, Qi Li, Yun-jian Wei, Yuan-hua Huang, Yan-lin Ma

**Affiliations:** 1 The First Affiliated Hospital of Zhengzhou University, Zhengzhou, 450052 Henan Province China; 2 Hainan Provincial Key Laboratory for Human Reproductive Medicine and Genetic Research, The First Affiliated Hospital of Hainan Medical University, Hainan Medical University, Hainan, China; 3 Department of Reproductive Medicine, The First Affiliated Hospital of Hainan Medical University, Hainan Medical University, Hainan, China; 4 Hainan Provincial Clinical Research Center for Thalassemia, The First Affiliated Hospital of Hainan Medical University, Hainan Medical University, Hainan, China; 5 Key Laboratory of Reproductive Health Diseases Research and Translation (Hainan Medical University, Ministry of Education, Hainan, China; 6 Haikou Key Laboratory for Preservation of Human Genetic Resource, The First Affiliated Hospital of Hainan Medical University, Hainan Medical University, Hainan, China

**Keywords:** Embryonic stem cells, Hematopoietic differentiation, OP9 cells, Co-culture

## Abstract

**Background:**

OP9 mouse stromal cell line has been widely used to induce differentiation of human embryonic stem cells (hESCs) into hematopoietic stem/progenitor cells (HSPCs). However, the whole co-culture procedure usually needs 14–18 days, including preparing OP9 cells at least 4 days. Therefore, the inefficient differentiation system is not appreciated. We aimed to optimize the culture conditions to improve differentiation efficiency.

**Methods:**

In the experimental group, we set six different densities of OP9 cells and just cultured them for 24 h before co-culture, and in the control group, OP9 cells were cultured for 4 days to reach an overgrown state before co-culture. Then we compared the hematopoietic differentiation efficiency among them.

**Results:**

OP9 cells were randomly assigned into two groups. In the experimental group, six different plated numbers of OP9 cells were cultured for 1 day before co-culture with hESCs. In contrast, in the control group, OP9 cells were cultured for 4 days at a total number of 3.1 × 10^4^ cells/cm^2^ in a 6-well plate to reach an overgrown state before co-culture. Hematopoietic differentiation was evaluated with CD34 immunostaining, and compared between these two groups. We could not influence the differentiation efficiency of OP9 cells with a total number of 10.4 × 10^4^ cells/cm^2^ in a 6-well plate which was cultured just for 1 day, followed by co-culture with hESCs. It reached the same differentiation efficiency 5 days earlier than the control group.

**Conclusion:**

The peak of CD34 + cells appeared 2 days earlier compared to the control group. A total number of 1.0 × 10^6^ cells in a 6-well plate for OP9 cells was appropriate to have high differentiation efficiency.

## Background

Human pluripotent stem cells (hPSCs), including human embryonic stem cells (hESCs) and induced pluripotent stem cells (iPSCs), have an inexhaustible capacity for self-renewal and differentiation into cells of the three germ layers. Therefore, PSCs are an attractive source for generating hematopoietic stem cells (HSCs) [1, 2], which are the core of hematopoiesis for their capability of cell proliferation and differentiation into different blood and immune cells [3, 4]. To date, allo-genetic HSC transplantation (allo-HSCT) is the only definitive cure for many malignant blood diseases, cancers, and genetic immune disorders. Primary HSCs are exclusively obtained from bone marrow, mobilized peripheral blood, and cord blood, however, the major problems are the limitation of HLA-matched donor sources and the difficulty of obtaining sufficient HSCs for transplantation, and hinder the use of stem cell therapy in the clinic. For the past several decades, methods have been developed to direct the differentiation of hPSCs to hematopoietic stem/progenitor cells (HSPCs) in vitro using either formation of the embryonic body (EB) [5], or co-culture with stromal cell lines that imitate hematopoietic microenvironment, such as OP9, S17, and AGM [6–8].

OP9 mouse stromal cell line has been widely used to induce hPSCs differentiation to HSPCs and multi-lineage hematopoietic cells [9–11]. One advantage of this co-culture system to induce hESCs differentiation into HSPCs is that it does not need additional cytokines. However, the disadvantage of this system is its low differentiation efficiency. The whole co-culture procedure usually needs 14–18 days, including preparing OP9 cells for at least 4 days. Previous studies optimized the OP9 co-culture system with a focus on adding extrinsic cytokines [10, 12] and stromal cell gene editing [9].

Traditional differentiation system does not set a precise density of OP9 cells for co-culture, i.e. overgrown OP9 cells were prepared by feeding and prolonged culture of confluent OP9 cells, followed by induction of hESCs differentiation into HSPCs for 10 days [6, 13]. We hypothesized that the density of stromal cells in a co-culture system can influence the efficiency of hematopoietic differentiation. In the present study, we examined the effects of different densities of OP9 cells in co-culture systems and determined the optimal density for efficient differentiation. We hope that this optimized system can be used as a new induction method for in vitro HSPC differentiation.

## Methods

### Cell culture

The hESCs line (HN14, 23–30 passages) was obtained from the Hainan Provincial Key Laboratory for Human Reproductive Medicine and Genetic Research and maintained on Matrigel-coated plates with hPSCs medium mTeSR™1 (Stem Cell Technologies, CAN) and passaged every 3 days at a 1:3 ratio using 0.5 mM EDTA, and changed fresh medium every day. Cells were maintained at 37 °C and 5% CO_2_ [14]_._

OP9 stromal cells (3–15 passages) were purchased from the Shanghai Cell Bank of the Chinese Academy of Sciences and cultured on 0.1% gelatinized plates in α-MEM (Gibco, USA) supplemented with 15% fetal bovine serum (FBS; Gibco, USA) until confluence. When passaged, these cells dissociated with 0.25% trypsin (Gibco) for 1 min at 37 °C. For cryopreservation, OP9 cells were suspended in 90% FBS added 10% Dimethyl Sulfoxide (Gibco) and stored in a deep freezer at -80 °C overnight, then transferred to liquid nitrogen.

OP9 cells and HN14 cells were co-cultured in a differentiation medium consisting of α-MEM medium supplemented with 10% defined fetal bovine serum (Hyclone, USA) and 100 μmol/L monothioglycerol (MTG; Sigma, USA) [9]_._

### Different co-culture strategies

OP9 cells were randomly assigned into two groups, experimental and control groups. In the experimental group, six different seeding numbers of OP9 cells were designed for each subgroup: 3.1 × 10^4^cells/cm^2^, 5.2 × 10^4^cells/cm^2^, 7.3 × 10^4^cells/cm^2^, 10.4 × 10^4^cells/cm^2^, 13.5 × 10^4^cells/cm^2^ and 16.6 × 10^4^cells/cm^2^ in 6-well plates. hESCs were plated on the related stromal cell layer (0.7–1.0 × 10^6^ cells in 6-well plate) [9]. These cells were cultured for 1 day and co-cultured with hESCs. In the control group, OP9 cells at a seeding number of 3.1 × 10^4^cells/cm^2^ in 6-well plates were cultured for 4 days to reach a confluent state followed by co-culture with hESCs. Undifferentiated hESCs were harvested by treatment with 2 mg/ml dispase enzyme (Gibco, USA) and scraped to maintain the cells in small clumps. The hESCs/OP9 cocultures were incubated for up to 10 days at 37 °C in normoxic conditions and 5% CO_2_ with a whole-medium change on day 1 and a half-medium change on days 4, 6, 8, and 10.

### Immunofluorescence staining

To determine the identity of cell line OP9, we examined the immunophenotype of OP9. They were negative for CD34 and uniformly positive for Calponin [15]. They were fixed with methanol-acetone (1:1) for 30 min at -20 °C. After washing in phosphate-buffered saline (PBS), cells were fixed with 0.05% Tween-20 and 10% goat serum in PBS for 1 h at room temperature, then incubated with primary antibodies against Calponin (Abcam, USA) and CD34 (Abcam, USA) overnight at 4 °C. Then they were washed 3 times and incubated with Donkey Anti-Rabbit IgG (H&L) (Abcam, USA) for an additional 60 min at room temperature. Finally, the cells were incubated with 4′6-diamidino-2-phenylindole (DAPI) for 20 min at room temperature.

### Flow cytometry analysis

To analyze the differentiation efficiency of the coculture system, hESCs/OP9 coculture cells were treated with 0.25% trypsin (Gibco, USA) for 20 min at 37 °C on days 8, 10 and 12. Trypsinization was terminated by adding a coculture medium. Co-culture cells were harvested by centrifugation, and washed twice by washing buffer (PBS supplemented by 5% FBS) [16]. These cells were immediately incubated with APC-human TRA-1–85 (R&D, USA), PE-Cy5.5 mouse anti-human CD34 (Becton Dickinson Company, USA) for 30 min at 4 °C. After staining, these cells were washed with washing buffer, centrifuged, and resuspended with moderate washing buffer, followed by flow cytometric analysis (BD FACSAria™ II sorter (BD Biosciences, USA). Data were analyzed with FlowJo version 7.6.1 (FlowJo, LLC). For negative control, APC mouse anti-human CD34 (Becton Dickinson Company, USA) was used to HN14, while OP9 cells labeled anti-CD34 [EP373Y] (Abcam, USA) and used Donkey Anti-Rabbit IgG (H&L) (Abcam, USA) as the secondary antibody.

### Magnetic sorting for CD34 positive cells

Single-cell suspension from day 10 of hESCs/OP9 co-cultures was labeled with CD34 Microbeads Kit (MACS) as recommended by the manufacturer and went through an LS separation column attached to a Midi-MACS Separator to obtain CD34^+^ cells. The purity of isolated CD34 + cells was always higher than 95% as evaluated by trypan blue exclusion.

### RNA isolation, RT-PCR, and quantitative RT-PCR

Total RNA was extracted from cultured cells using TRIzol reagent (Invitrogen, USA). cDNA was synthesized using 1 μg of RNA from each sample with iScript™ cDNA Synthesis Kit (Promega, USA). RT-PCR cycling conditions were as follows: initial denaturation at 95 °C for 10 min followed by 40 denaturation cycles at 95 °C for 15 s, primer annealing at 60 °C for 60 s, primer extension phase at 72 °C 15 s and final extension step at 72 °C for 7 min (Agilent Stratagene Mx3000P, Agilent Technologies, USA). GAPDH was used as an internal control. All experiments were performed three times. Sequences of primers used in this study are shown in Table 1.

**Table 1:** Primer sequences for RT-PCR

Gene	Forward (5’ to 3’)	Reverse (5’ to 3’)
GAPDH	CGAGATCCCTCCAAAATCAA	TGTGGTCATGAGTCCTTCCA
CD31	CCAAGGTGGGATCGTGAGG	TCGGAAGGATAAAACGCGGTC
CD34	CTACAACACCTAGTACCCTTGGA	GGTGAACACTGTGCTGATTACA
CD43	TCAGCCCTACCTCCCTCAAC	TATGGTTCCACCTGTCACGGT

### Colony-forming assay

Sorted CD34^+^ cells were plated at a density of 2.5 × 10^4^ cells/dish and suspended in 1 ml methylcellulose medium (MethoCult H4434, Stem Cell Technologies) in 35 mm low adherent dishes at 37 °C in a humidified atmosphere of 5% CO_2_ for 14–16 days. All clonogenic progenitor assays were performed in duplicate. Colonies of more than 50 cells were scored as positive clones and defined as macrophage (M), granulocyte and macrophage (GM), granulocyte (G), erythroid (E) according to their colony morphology.

### Statistical analysis

Data were analyzed with Prism 5 software (GraphPad, USA), and are presented shown as the mean ± SD. Significant differences were considered at the *P* < 0.05 level.

## Results

### Identification of OP9 cells and differentiation of hESCs

**Fig. 1:**
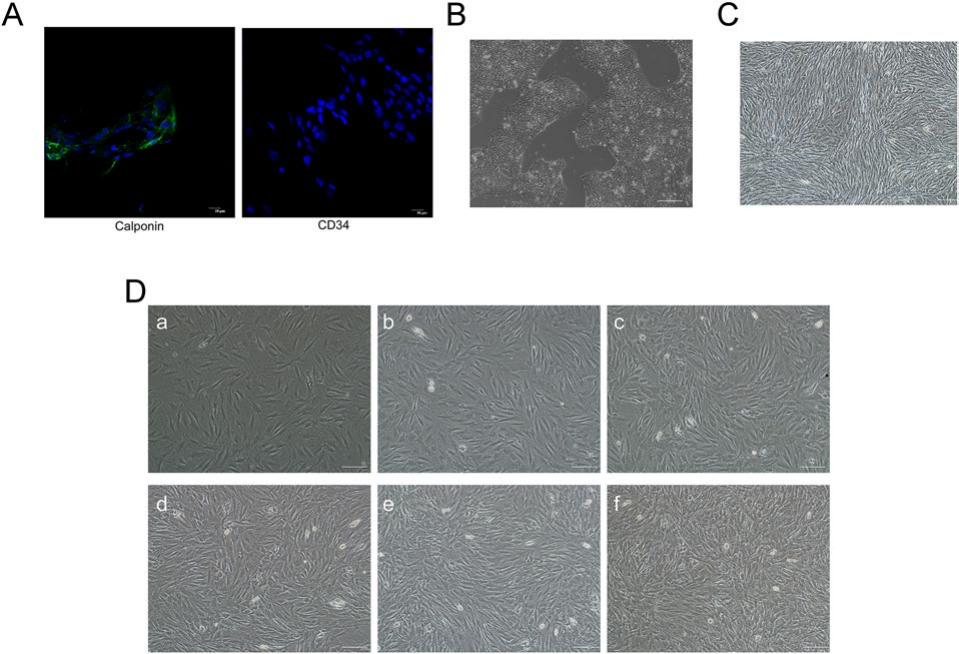
Identification and morphology of OP9 cells and hESCs before co-culture. **A** Immunophenotype of OP9 cells. Immunofluorescence staining of Calponin and CD34 in the OP9. Original magnification: × 40. **B** The morphology of hESCs (HN14) before co-culture (bar = 100 μm). hESCs monolayers at 80% confluence and co-cultured with OP9 cells. **C** The morphology of OP9 cells in the control group. OP9 cells reached confluence for culturing for 4 days at a seeding number of 3.1 × 10^4^ cells/cm^2^ in 6-well plates (bar = 100 μm). **D** The morphology of OP9 cells in the experimental group. OP9 cells were cultured for 1 day with six different seeding numbers in 6-well plates: a to f show the morphology of different seeding densities, i.e. 3.1 × 10^4^ cells, 5.2 × 10^4^ cells, 7.3 × 10^4^ cells, 10.4 × 10^4^ cells, 13.5 × 10^4^ cells, and 16.6 × 10^4^ cells per square centimeter, respectively (bar = 100 μm). When the seeding number of OP9 cells could reach 16.6 × 10^4^cells/cm^2^, the cultures reached confluence

**Fig. 2:**
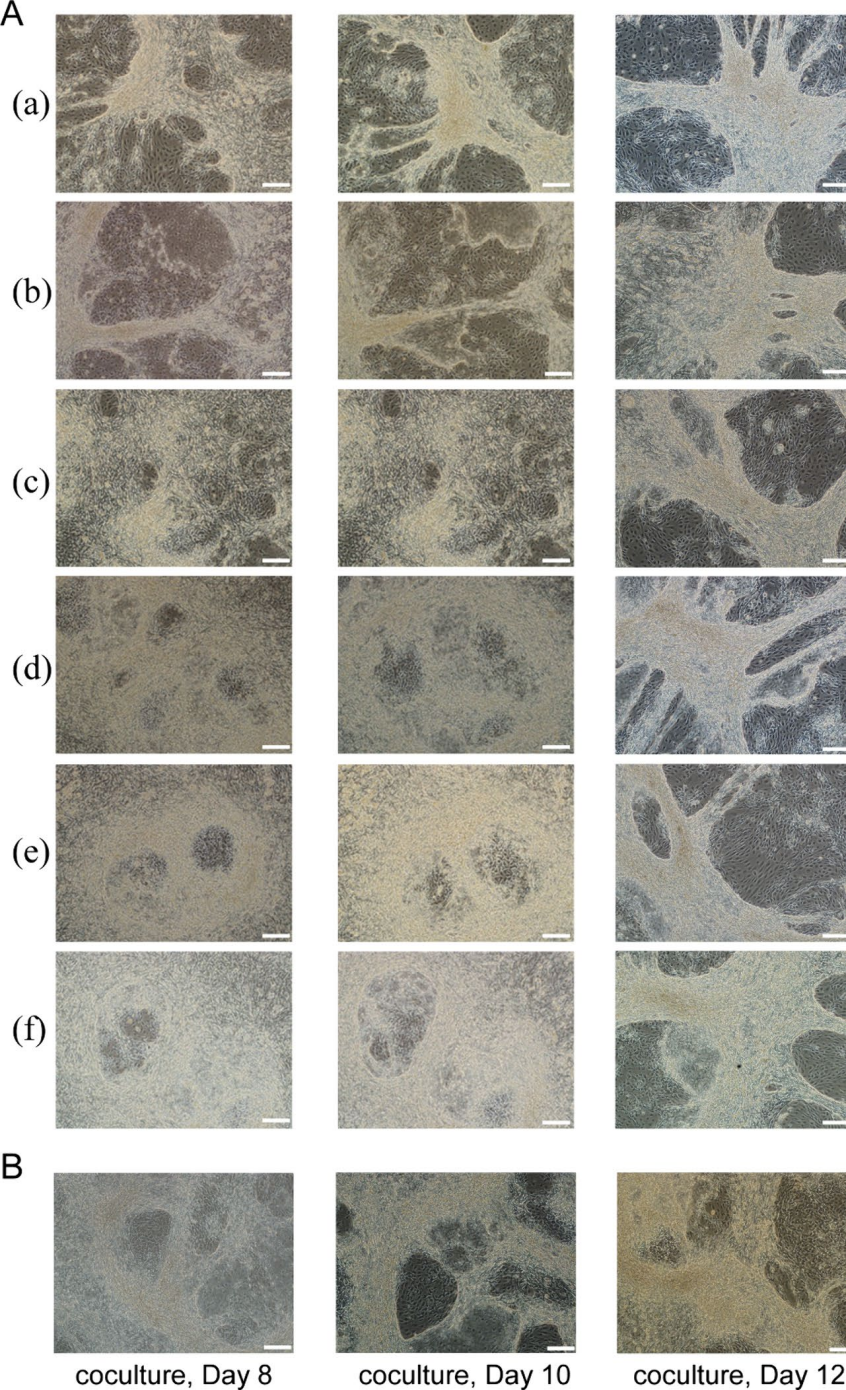
The morphology of ES cells on OP9 stromal cells during differentiation on days 8, 10 and 12. (**A**) a to f show the morphological changes of HN14 cells on six different seeding numbers of OP9 cells, i.e. 3.1 × 10^4^ cells, 5.2 × 10^4^ cells, 7.3 × 10^4^ cells, 10.4 × 10^4^ cells, 13.5 × 10^4^ cells, and 16.6 × 10^4^ cells/cm^2^, respectively, on days 8, 10 or 12 (bar = 100 μm). **B** The morphologies of differentiated HN14 colonies during co-culture on days 8, 10 and 12 in the control group (bar = 100 μm). In all groups, the presence of rounded cells at the edge of the colonies

The OP9 cells were bipolar fibroblast-like in morphology. They were morphologically homogeneous populations. Immunofluorescence staining was used to assess the molecular characteristics of the OP9 (Fig. 1A), the OP9 cells were negative for CD34 and expressed a marker of Calponin. As shown in Fig. 1D, OP9 cells were cultured for 1 day with six different seeding numbers in 6-well plates: a to f were the morphology of different seeding numbers,3.1 × 10^4^cells/cm^2^,5.2 × 10^4^cells/cm^2^,7.3 × 10^4^cells/cm^2^,10.4 × 10^4^cells/cm^2^,13.5 × 10^4^cells/cm^2^and 16.6 × 10^4^cells/cm^2^, respectively. OP9 cells plated at the numbers of 1.0–1.3 × 10^6^ cells reached nearly 100% confluency after 1 day of culture (d, e). When the seeding number of OP9 cells could reach 16.6 × 10^4^cells/cm^2^, the cultures reached confluence. In the control group, overgrown OP9 cultures were prepared for 4 days (Fig. 1C). HN14 cells were plated on the stromal cell layer when they reached 80% confluency. During the differentiation process, the morphology of cells started to change on days 8, 10, and 12 and hESCs started differentiating through the morphologic observation (Fig. 1B). As shown in Fig. 2, HN14 cells seeded on six different numbers of OP9 cells, 3.1 × 10^4^ cells, 5.2 × 10^4^ cells, 7.3 × 10^4^ cells, 10.4 × 10^4^ cells, 13.5 × 10^4^ cells, and 16.6 × 10^4^ cells per square centimeter, respectively, on days 8, 10 or 12 (bar = 100 μm).

### Effects of seeding density on the expression of CD34, CD43, and CD31

**Fig. 3:**
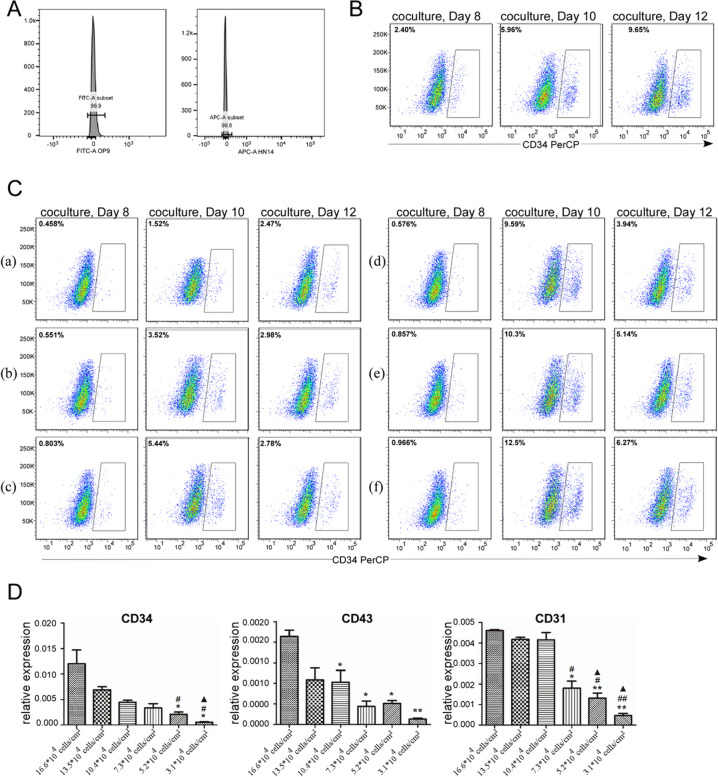
Determination of differentiation efficiency in the hESCs/OP9 co-culture system. **A** The expression of CD34 in undifferentiated human embryonic stem cells (HN14) and OP9 cells, separately. **B** Percentage of CD34^+^ cells by flow cytometry (x-axis) in the control group. The peak of hematopoietic differentiation on D12. **C** Percentage of CD34^+^ cells by flow cytometry (x-axis) in the experimental group. a to f show six different seeding numbers in 6-well plates, 3.1 × 10^4^ cells, 5.2 × 10^4^ cells, 7.3 × 10^4^ cells, 10.4 × 10^4^ cells, 13.5 × 10^4^ cells, and 16.6 × 10^4^ cells/cm^2^, respectively. Single-cell suspension from hESCs/OP9 co-culture obtained at the indicated time points was labeled with CD34 (x-axis). The number indicates the percentage of positive cells in the corresponding group. Except for (a) group, the peak of hematopoietic differentiation on D10. **D** Evaluation of the expression of hematopoietic-related genes on day 10 in the experimental group. RNA was extracted, and gene expression was quantified by qRT-PCR. Statistical results of phase-specific genes are shown as the mean ± SD, n = 3. ##* P* < 0.01 and #* P* < 0.05 compared with the 16.6 × 10^4^ cells/cm^2^ group. ▲▲* P* < 0.01 and ▲* P* < 0.05 compared with the 13.5 × 10^4^ cells/cm^2^ group. ★★* P* < 0.01 and ★* P* < 0.05 compared with the 10.4 × 10^4^ cells/cm^2^ group

Using flow cytometry analysis, we could easily distinguish human cells from OP9 because of APC-human TRA-1–85. CD34 is an important surface marker for HSPCs and can define HSPCs in the human hESCs/OP9 co-culture system [15]. CD34 was not expressed in original cells including HN14 and OP9 cells (Fig. 3A). During differentiation, we harvested co-culture cells on days 8, 10 and 12, and analyzed the expression of CD34 by flow cytometry. As shown in Fig. 3C, a to f were the six different seeding numbers of OP9 cells from 3.1 × 10^4^ cells to 16.6 × 10^4^ cells per square centimeter in 6-well plates. With the increasing density of OP9 cells, the efficiency of hematopoietic differentiation was improved from 2.47% to 6.27%. The group of seeding number of 10.4 × 10^4^cells resulted in 10% of CD34^+^ cells or even more on day 10. Except for the seeding number of 3 × 10^5^cells in a 6-well plate, all other groups had a peak of CD34^+^ cells on day 10 during hematopoietic differentiation. However, the control group had a peak of CD34 + cells on day 12 or later (Fig. 3B). These findings suggested that optimizing the seeding density of OP9 cells improves hESCs hematopoietic differentiation. Consistent with the results of flow cytometry, the expression of CD34 mRNA between 10,4 × 10^4^cells, 13.5 × 10^4^ cells, and 16.6 × 10^4^ cells/cm^2^ groups was comparable (Fig. 3D).

We also investigated the expression of CD31 and CD43 in these cells on day 10 (Fig. 3C) by qRT-PCR. CD43 and CD31 were also markers of HSPCs [3]. When the seeding number was up to 16.6 × 10^4^ cells per square centimeter in a 6-well plate, the expression of CD43 was increasing. We also found that when the seeding number was 10.4 × 10^4^ cells per square centimeter in a 6-well plate, CD31 expression increased more obviously than other densities. Likewise, the expression level of CD43 and CD31 reached a peak on day 10 in the experimental group.

### No effect of seeding density on the differentiation of HSPCs

**Fig. 4:**
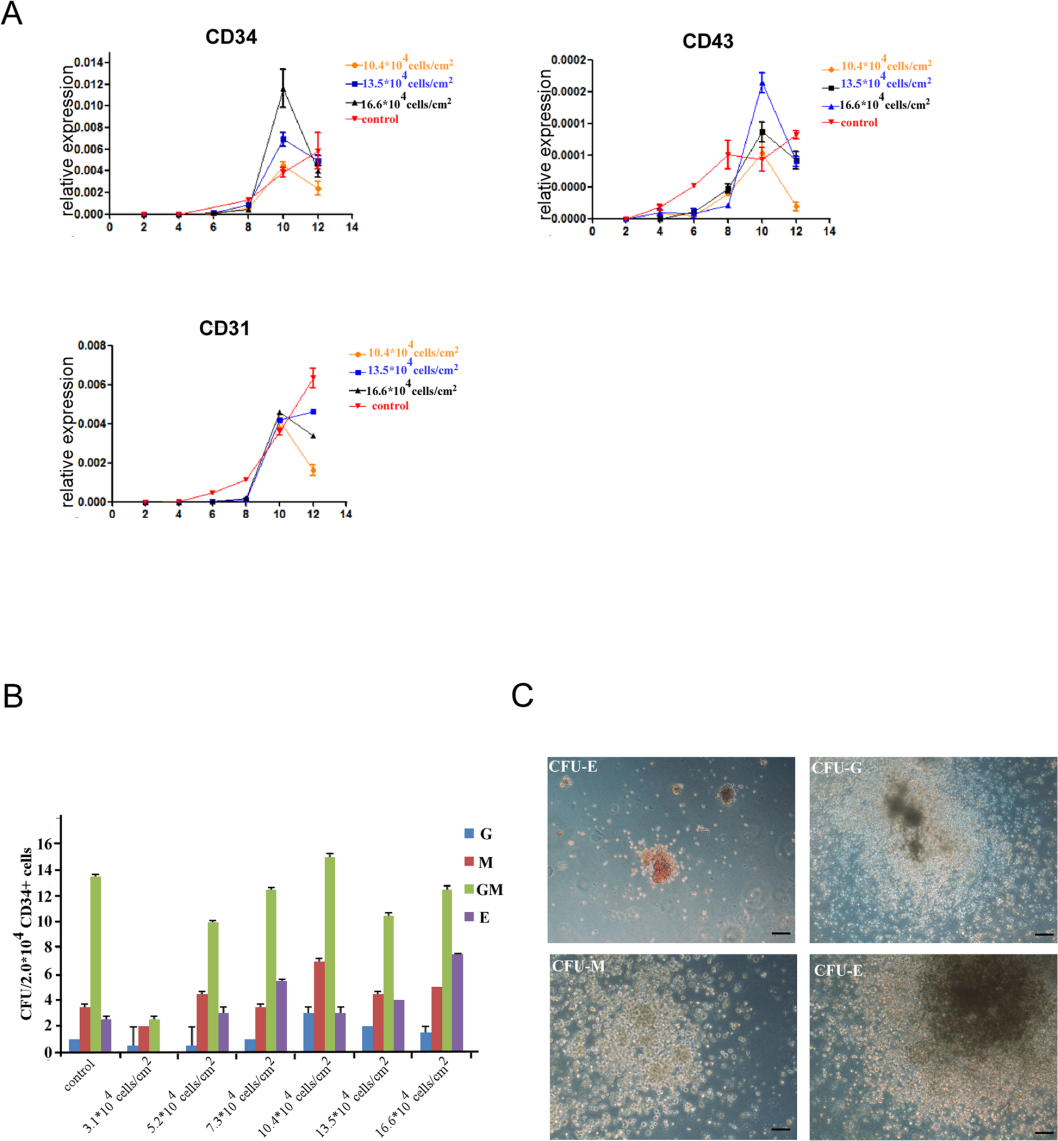
Improving the initial seeding number of OP9 cells shortened the whole time of co-culture system without changing the efficiency of hematopoietic differentiation. **A** Comparison of the expression of hematopoietic-related genes during differentiation between the experimental subgroup (10.4 × 10^4^ cells, 13.5 × 10^4^ cells, and 16.6 × 10^4^ cells/cm^2^ in a six-well plate) and the control group. Data are presented as means ± SD (n = 3). These results were consistent with flow cytometry. **B** Cell distribution in CFU from sorted CD34 positive cells for culturing 14–16 days in a Semi-solid medium. E, erythroid; G, granulocyte; GM, granulocyte/macrophage; M, macrophage. **C** Morphology of different colony-forming unit types, including M, GM, G and E. Scale bar = 100 mm

The purpose of this study was to optimize the co-culture system. We compared the expression of CD34, CD31, and CD43 between the experimental group on day 10 and the control group on day 12. The expression of CD34, CD31 and CD43 had no significant difference (Fig. 4A). We enriched CD34 + cells with magnetic cell sorting (Fig. 4B and C) and promoted them to differentiate into different CFUs, such as macrophage (M), granulocyte and macrophage (GM), granulocyte (G), erythroid (E). Consistent with the observations from flow cytometry and RT-qPCR, the proficiency of further differentiation had no difference between the two groups when the seeding density reached 5.0 × 10^5^cells/ml.

## Discussion

Stromal cells are one of the most important cell types in bone marrow that contain the hematopoietic niche, and hematopoiesis is regulated by microenvironment or niche [17, 18]. Some studies attempted to derive robustly induced HPCs from PSCs in vitro by simulating hematopoietic niches [18–20], and others tried to improve techniques to mimic the hematopoietic niche. The objective of these studies was to improve HSC differentiation to a large scale so that it may be used in regenerative medicine applications.

The co-culture technique was used for many applications, and the efficiency of differentiation was closely related to the condition of the stromal cells. Stromal cells producing M-CSF induce the differentiation of embryonic stem cells (ESC) down the monocyte-macrophage lineage [20, 21]. Nevertheless, OP9 stromal cells lacking M-CSF promote diferentiation down other haematopoietic lineages [6, 22, 23].

OP9 cells are one of the stromal cells that have been widely used because they provide more efficient support of hematopoiesis than other stromal cell lines probably due to that OP9 could release paracrine mediated signaling [24, 25]. In previous studies, hPSCs seeded onto overconfluent OP9 stroma, after 12 days of co-culture, with OP9 cells, the percentage of CD34 positive cells reached 10.3 ± 1.8%, but did not define the seed density and culture time before co-culture [26]. As the efficiency of co-culture was not stable, most of the studies focused on modification of gene expression, the function of OP9 stromal cells, or add hematopoietic-related cytokines to improve differentiation efficiency, previous studies showed that over-expressed transcription factor Lhx2 onto OP9 cells [9] or modified extrinsic factors directly on OP9 cells [27] enhanced hPSCs hematopoietic differentiation. With an in-depth understanding of hematopoietic differentiation, OP9 cells were modified with Notch ligand delta-like 1 to develop T lymphocytes [28] and NK cells [29]. Importantly, OP9 cells secreted factors and signaling molecules to induce hematopoietic differentiation [30, 31]. One study used single-cell q-PCR to track the dynamic gene expression of OP9-hESCs co-culture system at different stages and transiently over-expressed specific transcription factors (TFs) at different stages [32] and showed that over-expression of these TFs mainly promoted differentiation at the initial stage. Another recent study used 3D cell culture by co-culture of OP9 cells, and provided a minimal, scalable, biomimetic in vitro model of hematopoietic differentiation in the absence of exogenous cytokines [33]. Although OP9 stromal cells are universally used in hematopoietic differentiation. The efficiency of HSPC generation is related to the density of OP9 cells, however, no study explored the association between the density of OP9 cells with hematopoietic differentiation. We suggest that the longer OP9 cells were cultured, the more aging OP9 cells would become, and aging OP9 cells are prone to fat degeneration and insufficiency of secreting cytokines.

**Fig. 5:**
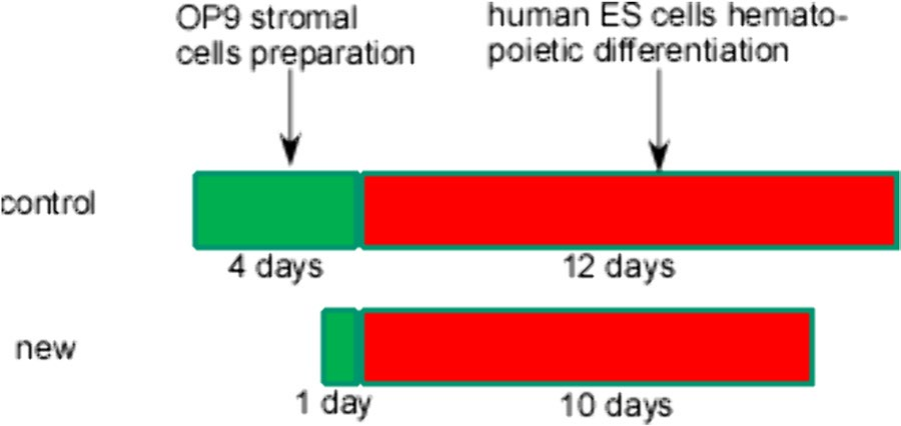
Schematic diagram showing two different differentiation systems. As shown in the schematic diagram, the optimized method could shorten the whole differentiation procedure by nearly 5 days

In the present study, to define the density of OP9 cells, we established an optimized OP9 co-culture system without adding any recombinant cytokines (Fig. 5). We focused on the seeding density of OP9 cells in the co-culture system. A seeding number of 16.6 × 10^4^ cells per square centimeter in a 6-well plate of OP9 cells was able to reduce culture time without significantly affecting the differentiation efficiency compared to previous study [26]. For example, the initial seeding density of OP9 cells at 10.4 × 10^4^ cells/cm^2^ in a 6-well plate had 10% percent of CD34^+^ cells at day 10, compared with day 12 in the control group. Increasing the density of OP9 cells further increased the percentage of CD34^+^ but showed no significant difference between the control and experimental groups. In other words, we reduced the differentiation time by 2 days with the same differentiation of efficiency. In addition, in our optimized OP9 co-culture system, HSPCs successfully differentiated into different CFUs (colony-forming units) with comparable efficiency, which meant that this system had the potential to produce multipotent hematopoietic progenitors. Thus, we have optimized the co-culture system to reduce the differentiation time for at least 5 days without changing differentiation efficiency.

## Conclusions

Our optimized approach is a novel, faster, and low-cost method to induce hematopoietic differentiation in vitro. However, the underlying mechanisms remain unclear. Future studies are needed to identify the possible factors or signaling molecules secreted by OP9 cells at different seeding densities and determine which is responsible for the improved differentiation observed in the present study. In the future, we will test related factors to induce differentiation and further optimize the differentiation system in the absence of OP9 cells.

## Data Availability

No datasets were generated or analysed during the current study.

## Abbreviations

hESC: Human embryonic stem cells
HSPC: Hematopoietic stem/progenitor cell
HPSC: Human pluripotent stem cells
iPSC: Induced pluripotent stem cell
EB: Embryonic body
TF: Transcription factors
CFU: Colony-forming units
